# Surrogate Indexes of Insulin Resistance in Dairy Goats: Transitional Variation in Subclinical Hyperketonemia

**DOI:** 10.3390/vetsci8060102

**Published:** 2021-06-06

**Authors:** Siqi Liu, Yezi Kong, Jing Wen, Yan Huang, Yaoquan Liu, Xiaoyan Zhu, Baoyu Zhao, Binyun Cao, Jianguo Wang

**Affiliations:** 1 College of Veterinary Medicine, Northwest A&F University, Yangling 712100, China; liusiqi647777@163.com (S.L.); kongyezi0207@163.com (Y.K.); hy040016@163.com (Y.H.); liuyq0703@163.com (Y.L.); xyzhu0922@nwsuaf.edu.cn (X.Z.); zhaobaoyu12005@163.com (B.Z.); 2 College of Animal Science and Technology, Northwest A&F University, Yangling 712100, China; wenjing3676@163.com

**Keywords:** dairy goat, insulin resistance, transition period, subclinical hyperketonemia, surrogate index

## Abstract

Background: Dairy goats are highly susceptible to subclinical hyperketonemia (SCHK) during the transition period. This study aimed to compare the variation in metabolic parameters and surrogate indexes of insulin resistance (sIR) between goats with SCHK and clinically healthy (HEAL) goats during the transition period. Methods: Twenty Guanzhong dairy goats were assorted to HEAL (*n* = 10) and SCHK (*n* = 10) groups according to the blood β-hydroxybutyrate (BHBA) concentrations. The blood samples were taken from the jugular vein of each goat at −3, −2, −1, 0 (partum), +1, +2, and +3 weeks relative to kidding to analyses GLU and INS. The sIR was calculated from blood metabolic parameters. Results: Compared with the HEAL goats, the insulin concentrations were significantly higher in SCHK goats during the first three weeks postpartum. The QUICKI, revised QUICKI (RQUICKI), and RQUICKI_BHBA_ were significantly lower in goats with SCHK at 1 week postpartum, while the homeostasis model assessment-IR (HOMA-IR) was significantly higher. Conclusion: Goats with SCHK made more efforts through elevated insulin levels at early lactation than HEAL goats, thereby maintaining the normal glucose concentrations.

## 1. Introduction

The transition period, ranging from 3 weeks prepartum to 3 weeks postpartum, is vital to the health status and reproductive performance of dairy goats [1]. During this period, fetal growth and milk secretion lead to a sharp increase of energy demands. As the rumen is squeezed by fetus, dry matter intake (DMI) is less than normal, leading to an inadequate energy intake [2]. Then, the negative energy balance (NEB) occurs when energy output overpasses input. The situation of energy deficiency motivates adipose tissue mobilization, resulting in elevated concentrations of nonesterified fatty acids (NEFA) and ketone bodies in the blood [3]. Hyperketonemia, which is defined as the increase of ketone bodies in the blood, is one of the metabolic disorders caused by NEB [4]. In dairy goats, hyperketonemia is also known as lactation ketosis or pregnancy toxemia, which usually happens in early postpartum or late pregnancy [5]. Subclinical hyperketonemia (SCHK) usually has no typical symptoms compared with clinical forms, but this metabolic disorder makes dairy goats more susceptible to other production diseases, such as mastitis and hypocalcemia [6]. Furthermore, SCHK is more common than clinical forms. The prevalence of SCHK of the Guanzhong goat in China is about 10% [7], thereby causing enormous economic losses to the dairy goat husbandry due to decreased milk production and SCHK-associated peripartal diseases.

Insulin resistance (IR) is a homeorhetic adaptation in ruminants for partitioning nutrients to support the uterus and mammary gland during the peripartum period [8]. Several surrogate indexes of IR (sIR) are developed to estimate INS sensitivity in human and veterinary medicine, which are calculated on the basis of blood NEAF, BHBA, GLU, and INS. These indexes include homeostasis model assessment-IR (HOMA-IR), quantitative INS sensitivity check index (QUICKI), revised quantitative INS sensitivity check index (RQUICKI), and revised quantitative INS sensitivity check index including β-hydroxybutyrate (RQUICKI_BHBA_) [9]. The sIR is convenient and simple to use compared with direct measurements of IR such as hyperinsulinemic-euglycemic clamp test (HEC), GLU tolerance test (GTT), and INS tolerance test (ITT) [10]. Several research studies have adopted sIR to estimate INS sensitive in dairy cows [11,12,13].

Underlying mechanisms of SCHK-induced insulin resistance is not yet unstudied in dairy goats. However, in dairy cows, many studies show a closed relationship between NEFA/BHBA with insulin resistance. An increased circulating NEFA concentration has been shown to cause an impaired insulin-stimulated glucose uptake by insulin-sensitive tissues [14]. A group of cows with high BHBA exhibited a higher insulin resistance, which was measured by GTT [15]. Another research study found that the elevated values of cortisol, insulin, NEFA, and BHBA are suggestive of impaired whole-body insulin sensitivity in SCHK cows during the transition period [16]. These data have demonstrated that there was a closed relationship between SCHK and IR, and IR plays a potential role in the development of SCHK. However, the relationship between IR and SCHK in dairy goats is not well understood. The purpose of this study was to compare the variations in sIR and metabolic parameters between dairy goats with SCHK and clinically healthy (HEAL) goats during the transition period.

## 2. Materials and Methods

### 2.1. Animals, Location, and Study Design

**Figure 1:**
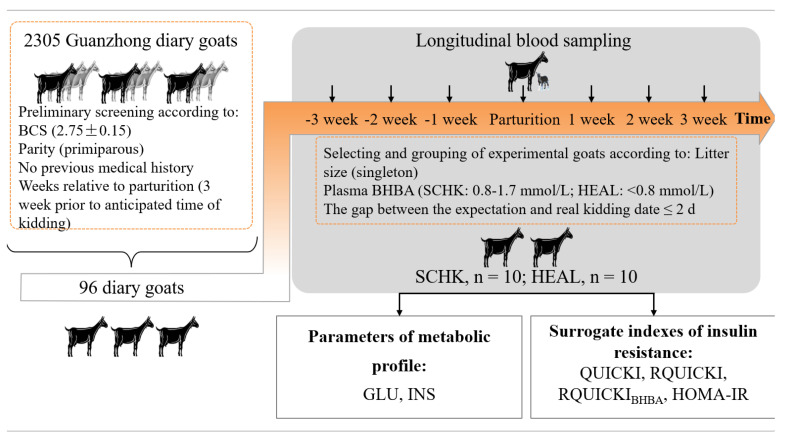
Study design. Two steps were used to screen and group animals. In the first step, 2035 Guanzhong dairy goats in the farm were subjected to estrous synchronization in September so that kidding occurred in February. Then, 96 Guanzhong dairy goats were enrolled and chosen from 2305 Guanzhong dairy goats according to BCS (2.75 ± 0.15), parity (primiparous), expected kidding date (within the first week of February), and no medical history. The blood samples were taken from the jugular vein of each goat at −3, −2, −1, 0 (partum), +1, +2, and +3 weeks relative to delivery, using vacutainer tubes with sodium heparin. The second step was to select these experimental goats by the litter size of singleton and allocate them to one of two groups according to their plasma BHBA concentrations, as either SCHK (*n* = 10; BHBA = 0.8–1.7 mmol/L) or HEAL (*n* = 10; BHBA < 0.8 mmol/L). Finally, the plasma biomarkers of metabolites were measured, and sIR were calculated.

The study was conducted in Western China (106°55′57″ E, 34°48′41″ N) at the experimental farm of Northwest A&F University (Shaanxi Province, China) between January and March in 2019. The protocol is schematically presented in Figure 1. Two steps were used to screen and group animals. In the first step, 2305 Guanzhong dairy goats on the farm were subjected to estrous synchronization in September so that kidding occurred in February. Then, 96 Guanzhong dairy goats were enrolled and chosen from 2305 Guanzhong dairy goats according to body condition score (BCS) (2.75 ± 0.15, mean ± SEM), parity (primiparous), expected kidding date (within the first week of February), and no medical history. The second step was to select these experimental goats by the litter size of singleton and allocate them to one of the following two groups according to their plasma BHBA concentrations: SCHK (*n* = 10; BHBA = 0.8–1.7 mmol/L) or HEAL (*n* = 10; BHBA < 0.8 mmol/L) [6,17,18]. The goats were reared in a free-stall barn from 3 weeks prior to the anticipated time of kidding until 3 weeks after kidding. All goats were fed the same diets comprising a base ration fed as a TMR, which was formulated to meet the nutrient requirements of dairy goats during the transition period according to the Nutrient Requirements of Small Ruminants (National Research Council, 2007). The goats had free access to water, and diets were fed ad libitum during the experimental period. The diet was offered twice daily at 0730 and 1530 h.

### 2.2. Plasma Samples and Laboratory Analyses

The blood samples were taken from the jugular vein of each goat at −3, −2, −1, 0 (partum), +1, +2, and +3 weeks relative to kidding, using vacutainer tubes with sodium heparin (Becton-Dickinson, Franklin Lakes, NJ, USA). Blood samples were collected before feeding in the morning prepartum, within 24 h after parturition, and after milking but before feeding in the morning postpartum. All the tubes were immediately placed on ice, centrifuged at 2000 *g* for 10 min, and stored at −80 °C until analyses of glucose (GLU), insulin (INS), BHBA, and NEFA concentrations.

Plasma concentrations of GLU (cat. No. GL8038, GOD-PAP method), BHBA (cat. No. RB1007, enzymatic method), and NEFA (cat No. FA115, colorimetric method) were analyzed using commercial kits (Randox Laboratories Limited, Crumlin, UK) and an automatic blood analyzer (Hitachi High-Technologies Corporation, Tokyo, Japan). Plasma INS concentrations were measured using a commercial goat INS enzyme-linked immunosorbent assay kit (cat No. MM-14170, Meimian Biotechnology, Yancheng, Jiangsu, China) and a microplate reader Bio-Rad 680 (Bio-Rad, Hercules, CA, USA) at 450 nm following the manufacturers’ protocols. The intra- and inter-assay coefficients of variation were 5.0 and 5.3%, respectively.

### 2.3. Surrogate Indexes for IR

The sIR, HOMA-IR, QUICKI, RQUICKI, and RQUICKI_BHBA_, was calculated using the following equations [9]:

HOMA-IR = [GLU (mmol/L) + INS (μIU/mL)]/22.5

QUICKI = 1/[lg GLU (mg/dL) + lg INS (μIU/mL)]

RQUICKI = 1/[lg GLU (mg/dL) + lg INS (μIU/mL) + lg NEFA (mmol/L)]

RQUICKI_BHBA_ = 1/[lg GLU (mg/dL) + lg INS (μIU/mL) + lg NEFA (mmol/L) + lg BHBA (mmol/L)]

### 2.4. Statistical Analysis

The data were statistically analyzed using GraphPad Prism 7.0 (GraphPad Software Inc., La Jolla, CA, USA). Repeated-measures analysis of variance, followed by Tukey’s multiple comparison test, was used to evaluate the differences in plasma metabolites and sIR. Repeated measures on each goat were taken into account (repeated factor: time during the transition period). The results were presented as mean ± SEM.

## 3. Results

**Figure 2:**
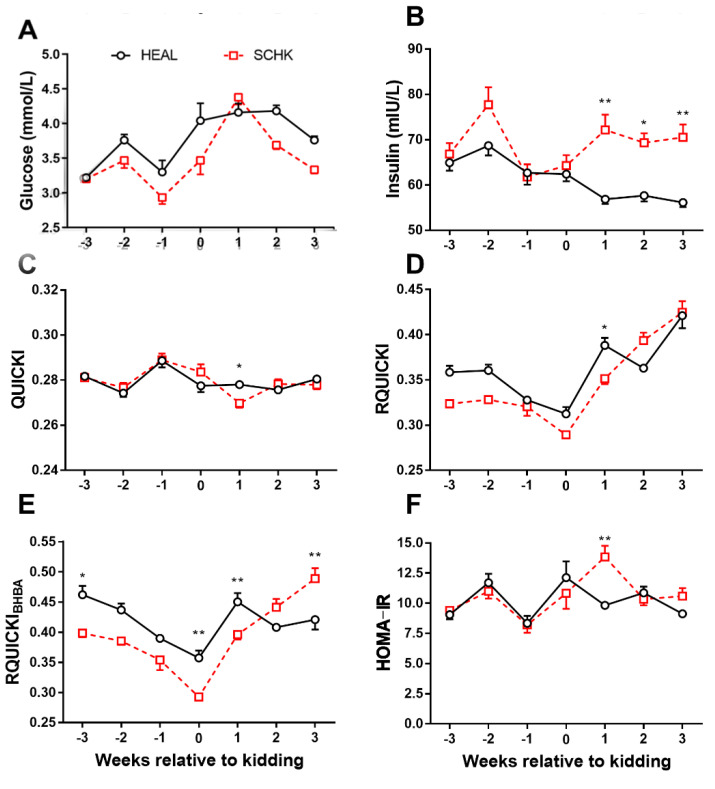
Changes in the concentrations of glucose (**A**), insulin (**B**), QUICKI (**C**), RQUICKI (**D**), RQUICKI_BHBA_ (**E**), and HOMA-IR (**F**) in goats with SCHK and HEAL goats during the peripartum period. * *p* < 0.05, ** *p* < 0.01. Values are presented as means ± SEM from 10 goats per group (*n* = 10).

The results of the present study showed that HEAL goats had slightly higher plasma GLU concentrations than SCHK except at 1 week postpartum (Figure 2A). The INS concentrations were significantly higher in goats with SCHK than in HEAL goats during early lactation (*p* < 0.05 or *p* < 0.01, Figure 2B). The QUICKI, RQUICKI, and RQUICKI_BHBA_ were significantly lower in goats with SCHK at 1 week postpartum than in HEAL (*p* < 0.05 or *p* < 0.01, Figure 2C–E). In contrast, The HOMA-IR was significantly higher in goats with SCHK at 1 week postpartum compared with the HEAL goats (*p* < 0.01, Figure 2F). The RQUICKI and RQUICKI_BHBA_ were the lowest at parturition.

## 4. Discussion

In the present study, we found that HEAL goats had slightly higher plasma GLU than SCHK. Another study found higher GLU concentrations in HEAL goats compared with multiparous goats with SCHK, which was consistent with the results of the present study [19]. The differences in the parity and severity of hyperketonemia might explain significant differences between the two studies. The lower GLU concentrations in SCHK were possibly a result of decreased endogenous GLU production, which was suppressed by high BHBA concentrations around parturition [2,20]. Unlike GLU, the INS concentrations were significantly higher in goats with SCHK than in HEAL goats during early lactation. This observation was consistent with a previous report in dairy cow [16]. It was speculated that INS secretion induced by elevated BHBA concentrations during lactation avoided further mobilization of adipose tissue, giving priority to energy for lactation [21]. The concentrations of INS in SCHK goats had significant increases after kidding. In our research, the plasma GLU in SCHK goats is just slightly lower than HEAL goats, but the plasma INS in SCHK goats is significantly higher than HEAL. That means that the large amounts of INS could not induce a comparable insulin-stimulated glucose utilization by skeletal muscle and adipose tissue. In other words, insulin induced a decreased biological response in insulin-sensitive tissues, which means insulin resistance.

Several surrogate indexes have been referenced as the evaluation indexes of INS sensitivity according to their importance in veterinary medicine [22]. The results of this study showed that the values of QUICKI, RQUICKI, and RQUICKI_BHBA_ were lower, while the value of HOMA-IR was higher, at 1 week postpartum in goats with SCHK compared with HEAL goats. It was concluded that goats with SCHK in this study exhibited significantly lower INS sensitivity at 1 week postpartum. However, estimating INS sensitivity using the aforementioned indexes in farm animals is still debatable. One study reported just two parameters of GTT, which are GLU clearance rate and fatty acids; area under the curve had weak correlations with IRs in periparturient dairy cows [23]. Another showed that IRs were not correlations with the parameters derived from the HEC in dairy cows at the end of the dry period [10]. More studies are needed to investigate the relationship between sIR and the parameters of direct measurements in order to determine the availability and effectiveness of sIR in dairy goats.

## 5. Conclusions

The results of the present study demonstrated that dairy goats with SCHK made more efforts to maintain the normal concentrations of GLU through elevated INS levels during the first 3 weeks of lactation.

## Abbreviations

SCHK	Subclinical hyperketonemia
HEAL	Health
HK	Hyperketonemia
BHBA	β-hydroxybutyrate
INS	Insulin
GLU	Glucose
NEFA	Non-esterified fatty acids
IR	Insulin resistance
sIR	Surrogate indexes of insulin resistance
QUICKI	Quantitative insulin sensitivity check index
RQUICKI	Revised quantitative insulin sensitivity check index
RQUICKI_BHBA_	Revised quantitative insulin sensitivity check index including β-hydroxybutyrate
HOMA-IR	Homeostasis model assessmentinsulin resistance
DMI	Dry matter intake
AT	Adipose tissue
BCS	Body condition score
NEB	Negative energy balance
HEC	Hyperinsulinemic euglycemic clamp test
GTT	Glucose tolerance test
ITT	Insulin tolerance test
